# An Engineered Fusion Protein Anti-CD19(Fab)-LDM Effectively Inhibits ADR-Resistant B Cell Lymphoma

**DOI:** 10.3389/fonc.2019.00861

**Published:** 2019-09-04

**Authors:** Dongmei Fan, Linlin Jiang, Yuewen Song, Shiqi Bao, Yuanyuan Yang, Xiangfei Yuan, Yongsu Zhen, Ming Yang, Dongsheng Xiong

**Affiliations:** ^1^State Key Laboratory of Experimental Hematology, Institute of Hematology and Hospital of Blood Diseases, Chinese Academy of Medical Sciences and Peking Union Medical College, Tianjin, China; ^2^School of Life Sciences, Ludong University, Yantai, China; ^3^Department of Oncology, Institute of Medicinal Biotechnology (IMB), Chinese Academy of Medical Sciences and Peking Union Medical College, Beijing, China

**Keywords:** adriamycin, BJAB cell line, BJAB/ADR cell line, engineered fusion protein, anti-CD19(Fab)-LDM

## Abstract

The 5-year survival rate of patients with B cell lymphoma is about 50% after initial diagnosis, mainly because of resistance to chemotherapy. Hence, it is necessary to understand the mechanism of chemo-resistance and to explore novel methods to circumvent multidrug resistance. Previously, we showed that an engineered cytotoxic fusion protein anti-CD19(Fab)-LDM (lidamycin), can induce apoptosis of B-lymphoma cells. Herein, we successfully established an adriamycin (ADR)-resistant B cell lymphoma cell line BJAB/ADR. The mRNA and protein level of ATP-binding cassette subfamily B member 1 (ABCB1) were significantly overexpressed in BJAB/ADR cells. Increased efflux function of ABCB1 was observed by analyzing intracellular accumulation and efflux of Rhodamine 123. The efflux of Rhodamine 123 could be significantly ameliorated by verapamil. Treatment with anti-CD19(Fab)-LDM at different concentrations induced cytotoxic response of BJAB/ADR cells similar to that of the sensitive cells. *In vivo* studies showed that anti-CD19(Fab)-LDM had better antitumor effect in BJAB and BJAB/ADR cell lymphoma xenografts compared with ADR or LDM treatment alone. Taken together, anti-CD19(Fab)-LDM can effectively inhibit the growth of BJAB/ADR cells both *in vitro* and *in vivo*. Anti-CD19(Fab)-LDM could be a promising molecule for the treatment of drug resistant cancers.

## Introduction

Lymphomas are a common heterogeneic group of hematologic diseases, among which B cell origin lymphoma represents the largest proportion (1, 2). At present, chemotherapy or chemoimmunotherapy remains the most effective therapeutic modality in the multifaceted treatment of lymphomas (3). Most patients who experience remission for more than 5 years have benefitted from the overall improvements in the treatment of B cell lymphomas. However, a significant portion of patients still show unfavorable response toward drug treatment. Currently, the clinical approaches to relapsed B lymphomas mainly involve in administering high-dose chemotherapeutic agents, using inhibitors to reverse drug resistance toward chemotherapy (4), or finding novel therapeutic strategies such as targeting CD20 or using Chimeric Antigen Receptor T-Cell Immunotherapy (CAR-T) (5–8). Multidrug resistance (MDR) or acquired chemo-drug resistance is a major contributor to the failure of chemotherapy as well as one of the major reasons for tumor relapse and metastasis (9–11). To investigate the mechanisms involved in the acquisition of chemotherapy resistance and subsequent poor prognosis, it is necessary to establish a proper resistant cell model derived from a drug-sensitive human lymphoma cell line. Adriamycin (ADR; generic name: doxorubicin, DOX) is a chemotherapeutic drug frequently used in multiple clinical protocols of chemotherapy and is also a critical drug in the treatment of lymphoma (12). Unfortunately, some lymphomas have shown ADR resistance with continued treatment (13, 14). Therefore, establishing an ADR-resistant lymphoma cell model is useful for studying the mechanism of resistance in B cell lymphoma and for searching solutions regarding ADR resistance.

Lidamycin (LDM), originally named C-1027, is a member of the enediyne antibiotic family with strong cytotoxic effect toward human cancer cells and its mechanism of action is related to DNA damage. Importantly, LDM molecule is composed of a highly active group enediyne chromophore (AE) and a protective group apoprotein (LDP) (15, 16). The non-covalent bond between AE and LDP can be dissociated and re-associated, leading to rebuilting a molecule that exhibits similar activity as that of natural LDM. Taking advantage of the LDP genetic reassortment and the specific targeting capability of antibody fragments, different types of engineered fusion proteins were created (17–20). In short, lidamycin can be linked with another component, such as antibodies, due to its unique structure. As a result, lidamycin can target a specific site with its cytotoxicity. CD19 is a biomarker that is expressed on virtually all neoplastic cells of the B-cell lineage (21, 22). Previous studies demonstrated that the engineered fusion protein anti-CD19(Fab)-LDM, which comprises the chemo-drug lidamycin and anti-CD19(Fab) antibody, showed targeted cytotoxicity against lymphoma cells both *in vitro* and *in vivo* (23).

In this article, to verify the anticancer activity of the engineered fusion protein anti-CD19(Fab)-LDM on multidrug-resistant cells, we established an ADR resistant lymphoma cell line BJAB/ADR. Furthermore, we showed that anti-CD19(Fab)-LDM engineered fusion proteins could target the cell surface marker CD19 and exert the same cytotoxicity effect on ADR-resistant BJAB cells as on BJAB-sensitive cells. Our study indicates that anti-CD19(Fab)-LDM has anticancer effects on ADR-resistant B cell lymphoma. This result sheds light on the therapeutic effect of this fusion protein and provides a promising solution for MDR, especially ADR-resistant B cell lymphoma.

## Materials and Methods

### Chemicals and Reagents

Adriamycin (ADR), propidium iodide (PI), verapamil and RNase A were obtained from Sigma-Aldrich Trading Co, Ltd (St. Louis, MO, USA). The phospho-glycoprotein (P-gp, MDR1) mouse monoclonal antibody conjugated with Alexa Fluor 594 (sc-390883), ABCG2 mouse monoclonal antibody conjugated with Alexa Fluor 488 (sc-18841) and MRP1 mouse monoclonal antibody conjugated with Alexa Fluor 488 (sc-53130) were obtained from Santa Cruz Biotechnology, Inc (Dallas, TX, USA). LDM was provided by the Institute of Medicinal Biotechnology of the Chinese Academy of Medical Sciences (Beijing, China). Antitumor agents were prepared fresh in PBS (phosphate-buffered saline) immediately prior to use.

### Cells and Cell Culture

Cell culture supplies, including Dulbecco's modified Eagle's Medium (DMEM), fetal bovine serum (FBS), penicillin/streptomycin and 0.25% trypsin, were purchased from Corning Incorporated (Corning, NY, USA). The BJAB cell line was obtained from Cell Resource Center, Institute of Hematology and Hospital of Blood Diseases, Peking Union Medical College (PUMC) (Beijing, China). The cells were cultured in RPMI 1640 medium supplemented with 10% fetal bovine serum (FBS) and 1% penicillin/streptomycin, and they are maintained in an incubator containing 37°C humidified air with 5% CO_2_.

### Establishment of an ADR-Resistant BJAB Cell Line

The ADR-resistant cell line was created from the BJAB parental cell line via intermittent exposure to increasing concentrations of ADR for 6 months. Briefly, BJAB/ADR cells were treated with ADR with the concentrations ranging from 37 nM to 294 nM in a stepwise increasing manner. At first, the majority of the cells died after being treated with low concentrations of ADR for 24 h. We used 0.01 mol/L PBS to wash the surviving cells and continued to culture them in ADR-free growth medium. When cells were in the logarithmic growth phase, they were exposed to a higher ADR concentration for 24 h. After this process was repeated in a stepwise manner, a single-cell-derived ADR-resistant subclone, designated as BJAB/ADR, was established. For the maintenance of MDR, BJAB/ADR cells were cultured with 147 nM ADR. Two weeks before the experiment, BJAB/ADR cells were maintained in drug-free culture medium and passaged at least 3 times.

### Cell Growth Assay

To investigate cell growth in both BJAB and BJAB/ADR cells, a cell proliferation assay was performed. Briefly, we seeded cells into 24-well culture plates at a density of 5 × 10^3^ cells per well and cultured in complete RPMI 1640 culture medium for 8 days. Trypan blue exclusion-based methods were used to determine cell counts, and cells from triplicate wells were counted every 24 h for 8 days. All experiments were independently performed three times.

### Analysis of Cell Cycle Distribution

After BJAB and BJAB/ADR cells were treated with ADR, they were harvested, washed twice with ice-cold PBS (pH 7.2), centrifuged and resuspended in 500 μL ice-cold PBS, and adjusted to a density of 1 × 10^6^ cells/mL. Then, the cells were fixed with 70% ethanol at −20°C overnight. For the next step, the cells were incubated with 100 μL RNase (100 μg/mL, Sigma) for half an hour and stained with 200 μL PI (50 μg/mL) for 1 h. Data from 100,000 events/sample were collected via FACScan flow cytometer (Becton Dickinson, San Diego, CA) and analyzed using FlowJo software.

### Cell Viability Analysis (MTT Assay)

The MTT colorimetric assay was used to determine cell viability. Briefly, BJAB or BJAB/ADR cells (approximately 6,000 cells/well) were seeded into 96-well plates one day before drug treatment. After 72 h of drug treatment, 20 μL MTT solution (5 mg/mL thiazolyl blue powder in PBS) was added into each well and further incubated for 4 h at 37°C in a humidified atmosphere with 5% CO_2_. At the end of the incubation period, the supernatant, including the medium and MTT solution, was removed from each well, and the forming formazan crystals were dissolved by adding 100 μL dimethyl sulphoxide (DMSO) solution and agitating the plate for 15 min. The spectrophotometric absorbance was measured at 570 nm. The percentage of viable cells was calculated compared with the untreated control group (assumed 100% viability). After treatment with ADR at different concentrations, the resistance fold was reflected by MTT colorimetric analysis. In a separate experiment, 10 μL verapamil (2.5 mg/mL) was used to treat BJAB/ADR cells to observe whether verapamil can reverse ADR-induced MDR. The cytotoxicity of anti-CD19(Fab)-LDM on cells was evaluated in the same way. All experiments were repeated three times independently.

### Primer Design

Primers were designed according to published sequences using web-based software. We used the “BLAST” program (http://www.ncbi.nlm.nih.gov/blast) to determine the specificity of the primers. The primers used in this study were as follows: GAPDH-F: GAAGGTGAAGGTCGGAGTC, GAPDH-R: GAAGATGGTGATGGGATTTC; and MDR1-F: CCCATCATTGCAATAGCAGG, MDR1-R: GTTCAAACTTCTGCTCCTGA.

### RNA Extraction

Total RNA was extracted from cells using a RNeasy Mini Kit (Qiagen) following the instructions of manufacturer. Then, cDNA was synthesized with an M-MLV Reverse Transcriptase Kit (Invitrogen™) following the manufacturer's instructions.

### Quantitative Real-Time PCR (qRT-PCR)

Quantitative Real-time PCR (RT-PCR) was used to quantitatively detect mRNA of *ABCB1, ABCC1*, and *ABCG2* in BJAB and BJAB/ADR cells. RT-PCR was performed using a SYBR® Green PCR Master Mix kit (Applied Biosystems®) on the Applied Biosystems 7500 system. The thermal profile comprised 40 cycles as follows: 95°C for 30 s, 55°C for 60 s, and 72°C for 30 s. The expression of each gene was normalized using the mean expression of the housekeeping gene. Linearized relative expression was obtained according to the 2^−ΔΔ*CT*^ method (24).

### Detection of MDR Protein Expression Level by Flow Cytometry

Control and ADR-resistant BJAB cells were harvested, washed twice with ice-cold PBS (pH 7.2) and placed on ice immediately after collection. Samples (50 μL) were stained at 4°C for 20 min using predetermined saturating concentrations of phycoerythrin (PE)-labeled anti-P-gp monoclonal antibody, fluorescein (FITC)-labeled anti-ABCG2 monoclonal antibody, or FITC-labeled anti-MRP1 monoclonal antibody, respectively. Cells were analyzed on a FACScan flow cytometer (Becton Dickinson, San Diego, CA). Positive and negative cell populations were determined by using unreactive isotype-matched mAbs (Coulter) as controls for background staining. Background levels of staining were delineated using gates established to include 98% of the control cells.

### Assessment of the Efflux Function of ABC Transporter in BJAB/ADR Cells

Because Rhodamine 123 (Rho 123) is a reference fluorescent substrate of ABCB1, we detected the fluorescence intensity to obtain efflux function of ABCB1 (25). Briefly, BJAB and BJAB/ADR cells were suspended at a density of 5 × 10^5^ cells/mL in serum-free RPMI 1640 medium, and 200 μL of the cell suspension was put into 1.5 mL microcentrifuge tubes. BJAB cells were divided into two groups: negative control group (PBS) and positive control group (Rho 123). BJAB/ADR cells were divided into three groups: negative control group (PBS), positive control group (Rho 123) and experimental group (verapamil plus Rho 123). In the experimental group, 50 μmol/L verapamil was added to the tubes and incubated for 30 min at 37°C. After incubation, Rho 123 (200 nmol/L) was added to each tube. The cells were incubated for 1 h and then washed twice with ice-cold PBS after the incubation period. Finally, the fluorescence intensity was determined by flow cytometry to measure intracellular accumulation and efflux of Rho 123. Data was obtained from FACScan flow cytometer (Becton Dickinson, San Diego, CA). All experiments were independently conducted three times.

### *In vivo* Antitumor Activity in Subcutaneous Xenograft Tumor Models

All experiments on mice received humane care in compliance with the Public Health Service Policy on Humane Care and Use of Laboratory Animals. The study protocol was approved by the Institutional Animal Care and Use Committee of the State Key Laboratory of Experimental Hematology (SKLEH).

BJAB and BJAB/ADR cells were harvested, suspended in PBS, and then subcutaneously injected into 5-week-old female BALB/c nude mice (1 × 10^7^ cells/0.2 mL/mouse) to establish the BJAB and BJAB/ADR xenograft tumor models. When tumor volumes reached 60–80 mm^3^, mice were randomized into eight treatment groups (five mice per group). Group 1: animals received PBS; group 2: animals received 6 nmol/kg ADR; group 3–5: animals received 2 nmol/kg, 4 nmol/kg, and 6 nmol/kg LDM, respectively; and group 6–8: animals received 2 nmol/kg, 4 nmol/kg, and 6 nmol/kg anti-CD19(Fab)-LDM, respectively. Drugs were intravenously injected once. The body weights of the mice and the two perpendicular diameters of the tumors were recorded every third day, and tumor volumes were calculated by the following formula: tumor volume = 1/2 × length × width^2^. Animals were sacrificed, and xenograft tumors were surgically dissected, weighed and measured 28 days after treatment initiation.

### Statistical Analysis

All the data was shown as the mean ± SD obtaining from three independent experiments performed in triplicate. The results were analyzed with one-way analysis of variance (ANOVA). Comparisons are made between control groups and corresponding treatment groups and they were carried out via SPSS 10.0 software. A value of P <0.05 was considered statistically significant.

## Results

### Successful Establishment of the ADR Resistant BJAB/ADR Cell Line

The BJAB/ADR cell line was established after intermittent treatment with ADR at concentrations ranging from 37 to 294 nM in a stepwise increasing manner. Over 6 months, a clone of BJAB cells that resistant to ADR was successfully screened and named BJAB/ADR. BJAB/ADR cells could grow sufficiently even if cultured in RPMI 1640 medium with 294 nM ADR, and cells also maintained resistance to ADR after removal of the drug for at least 2 weeks. Normally, the ADR-resistant cells were maintained in complete culture medium with 147 nM ADR, which is the approximate IC_50_ value (concentration that reduces viability to 50%) of BJAB/ADR cells to ADR. Moreover, BJAB/ADR cells can stably grow in drug-free RPMI 1640 medium for more than 2 weeks. These results suggest that an ADR-resistant cell line was successfully established.

**Figure 1:**
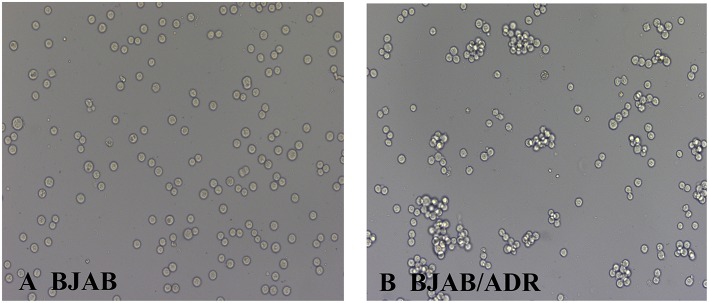
Morphological characteristics of BJAB and BJAB/ADR cells. BJAB cells **(A)** and BJAB/ADR cells **(B)** were observed under an optical microscope (original magnification × 200). Compared with BJAB cells, BJAB/ADR cells tended to grow in clusters.

In addition, the morphological characteristics of the established ADR-resistant BJAB/ADR cells were distinct from those of its parental cells under optical microscope. Although both types of cells exhibited suspension growth and had relatively consistent size and shape, the BJAB parental cells grew as a monolayer (Figure 1A), while the BJAB/ADR resistant cells tended to grow in clusters (Figure 1B).

### BJAB/ADR Cells Had a Slower Growth Rate Than the Parental Cells and Were Arrested in G0/G1 Phase

**Figure 2:**
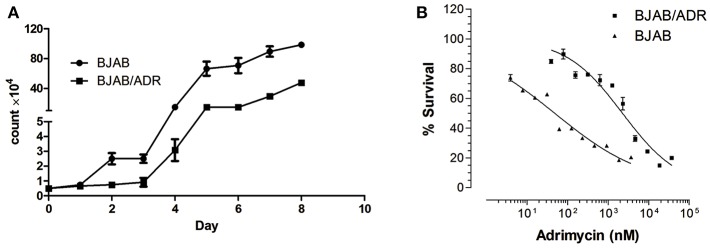
Cell growth curve of BJAB and BJAB/ADR cells. **(A)** Growth curves of BJAB and BJAB/ADR cells. BJAB/ADR cells grew slower than BJAB cells (*P* < 0.05). **(B)** Cytotoxicity of adriamycin against BJAB and BJAB/ADR cells. Each data point was obtained from three independent experiments in triplicate. BJAB/ADR cells were resistant to adriamycin.

The growth curves of BJAB and BJAB/ADR cells are shown in Figure 2A. The proliferation rate of both cell lines was not significantly different when the cells were cultured at low density. However, the growth rate of BJAB cells increased much more quickly as the density increased (*P* <0.05). Specifically, the cell population doubling times for BJAB and BJAB/ADR cells were 31.66 ± 1.2 h and 35.19 ± 2.1 h, respectively (*P* <0.05).

To investigate the effect of ADR on cell growth, a cell cycle assay was performed via flow cytometry. The results showed that the proportion of BJAB/ADR cells in the G0/G1 phase increased (*P* < 0.05, Table 1) and was accompanied by a decreased proportion in the S phase and G2/M phase. These results indicated that ADR could induce G0/G1 phase arrest in BJAB/ADR cells compared with the phase distribution of BJAB cells. However, the difference of phase distribution between BJAB and BJAB/ADR was not obvious, even they were repeatable and statistically significant. Hence, BJAB/ADR cell line may involve in other mechanisms of action resulting in ADR-resistant, which is needed to be addressed further.

**Table 1:** Cell cycle distribution of BJAB and BJAB/ADR cells.

**Cell line**	**Cell cycle phase**		
	**G0/G1 (%)^b^**	**S (%)**	**G2/M (%)**
BJAB	30.28 ± 0.36	5.98 ± 0.422	63.73 ± 0.537
BJAB/ADR	34.03 ± 0.068^*^^a^	4.16 ± 0.18^*^	61.81 ± 0.12

a* P <0.05, the values are shown as the mean ± SD obtained from three independent assays.

b *The increased proportion of resistant BJAB/ADR cells in G0/G1 phase was accompanied by a decreased proportion of cells in S and G2/M phases*.

### BJAB/ADR Cells Exhibited a 43-fold Greater ADR Resistance Level Than the Parental Cells and Showed Cross-Resistance to Other Anticancer Drugs

After cells were treated with ADR, we performed the MTT assay to determine the drug resistance factor (RF). The IC_50_ values of ADR for BJAB and BJAB/ADR cells were 57.156 ± 2.30 nM and 2,434 ± 111.476 nM (Figure 2B), respectively. As shown in the results, the ADR resistance level of BJAB/ADR cells was 43-fold greater than that of the parental cells. This result verified that the BJAB/ADR cell line acquired ADR resistance.

Additionally, BJAB/ADR cells showed resistance to various structurally unrelated anticancer drugs other than ADR. The cross-resistance profile of BJAB/ADR was summarized in Table 2. BJAB/ADR cells showed strong cross-resistance to etoposide, daunorubicin, homoharringtonine, and mitoxantrone but not cisplatin. Interestingly, the ADR-resistant subclone was 40 times more resistant to daunorubicin than the parental cell line. Hence, the established BJAB/ADR cell line can also be used for MDR study of its substrate drugs.

**Table 2:** Cross-resistance profile of BJAB/ADR cells to other anticancer drugs.

**Treatment**	**IC**_****50****_**(mean** **±** **SD)**^a^		**RF^b^**
	**BJAB (nM)**	**BJAB/ADR (nM)**	
Adriamycin	57.16 ± 2.30	2,434.12 ± 111.48	43
Etoposide	680.53 ± 21.11	21,200.22 ± 0.47	31
Cisplatin	*6, 772.20*±375.38	7,068.27 ± 435.44	1
Daunorubicin	277.09 ± 39.02	11,100.05 ± 0.14	40
Homoharringtonine	73.34 ± 20.11	568.23 ± 22.67	8
Mitoxantrone	75.36 ± 6.93	2,900.59 ± 410.21	38

a IC_50_ values are shown as the mean ± SD calculated from the results of at least three independent MTT assays.

b *RF refers to the resistance factor, which was calculated by dividing the IC_50_ values of the resistance cell line by the IC_50_ values of the respective parental cell line*.

### The Expression of Both *ABCB1* Gene and P-gp Protein Increased in BJAB/ADR Cells

Since it is reported that ADR is transported by ATP-binding cassette (ABC) transporters, especially ABCB1 and ABCG2 (4, 11), we hypothesized that the resistance mechanism of BJAB/ADR cells was associated with overexpression of ABC transporters. The *ABCB1* gene is a member of the ABC transporter superfamily that encodes a 170-kDa plasma membrane ABCB1 (P-glycoprotein, P-gp). ABC transporter functions as a drug efflux pump, thus resulting in decreased intracellular concentrations of broad drugs, such as paclitaxel, doxorubicin and others (26).

**Figure 3:**
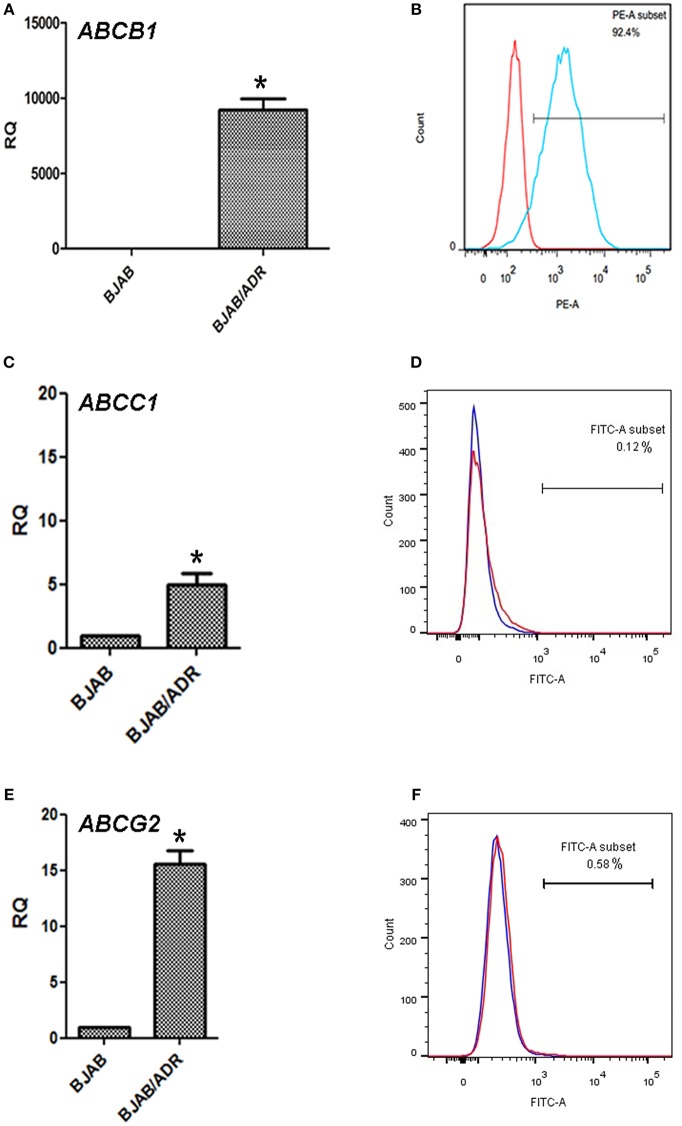
Expression of the *ABCB1, ABCC1, ABCG2* genes and P-gp, MRP1, BCRP proteins in BJAB/ADR cells. **(A)** The expression of the *ABCB1* gene in the ADR-resistant BJAB/ADR cells. The mRNA level of MDR1 analyzed by qRT-PCR and normalized to the mRNA level of the housekeeping gene GAPDH. Compared with the sensitive cells BJAB, BJAB/ADR drug-resistant cells showed increased expression of *ABCB1* mRNA. ^*^P < 0.05, compared with control group. **(B)** The protein expression of P-gp on BJAB/ADR cells. The expression of P-gp was evaluated via flow cytometry analysis. BJAB/ADR cells showed higher expression of the membrane protein P-gp compared with the BJAB sensitive cells. **(C,E)** The qRT-PCR on the gene expression of *ABCC1* and *ABCG2*, respectively. These results showed higher expression levels of both genes, but the increased expression was not as high as that of *ABCB1*. ^*^P < 0.05, compared with control group. **(D,F)** The protein expression level of MRP1 and BCRP, separately. The results were obtained from flow cytometry analysis. These results indicated that higher expression levels of both protein, but the increased expression was not as high as that of P-gp.

To determine the underlying resistance mechanism of BJAB/ADR cells, qRT-PCR was performed to detect the expression of the *ABCB1, ABCC1*, and *ABCG2* genes. We found that the *ABCB1* gene was upregulated in BJAB/ADR cells (*P* < 0.05) (Figure 3A). Moreover, the expression of P-gp protein was evaluated by flow cytometry analysis. Compared with the BJAB-sensitive cells, BJAB/ADR cells showed higher protein expression level of P-gp (*P* < 0.05) (Figure 3B). The results were consistent with those results from the qRT-PCR assay. We also examined the mRNA level of *ABCC1* and *ABCG2*, plus protein expression level of MRP1 (multidrug resistance-associated protein 1) and BCRP (breast cancer resistance protein), as shown in Figures 3C–F. Both transporters showed increased mRNA and protein expression, but not increased as much as *ABCB1* and P-gp expression. These results implicated that these two transporters were probably related to cross-resistance or MDR, suggesting that the mechanism of drug resistance of BJAB/ADR cells might be due to the overexpression of ABCB1.

### Overexpression of ABCB1 in BJAB/ADR Cells Increased Drug Efflux, and Verapamil Reversed the Chemoresistance of the Cells to Adriamycin

**Figure 4:**
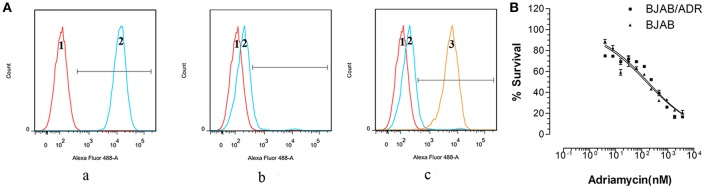
Intercellular accumulation and efflux of Rho 123. **(A)** Flow cytometry measured intracellular accumulation and efflux of Rho 123. (a) Treatment of BJAB cells with Rho 123 resulted in Rho 123 accumulation in parental cells. (b) Decreased fluorescence intensity of Rho 123 in BJAB/ADR cells compared with that in the BJAB cells. (c) The chemo-drug accumulation significantly increased after BJAB/ADR cells treated with verapamil, an inhibitor of ABCB1 transporter, for 1 h (1 represents negative cells, 2 represents positive cells and 3 represents BJAB/ADR positive cells treated with verapamil). **(B)** Verapamil reversed the chemoresistance of BJAB/ADR cells to adriamycin, thus increasing the sensitivity of the drug-resistant cells to adriamycin.

As shown above, ABCB1 was overexpressed in the ADR-resistant cell line. To further understand the effects of ABCB1 overexpression on drug resistance, we performed an accumulation and efflux assay using Rho 123, a reference fluorescence substrate of ABCB1, via flow cytometry (27). As Figure 4Aa,b shown, the mean values of the fluorescence intensity in BJAB and BJAB/ADR cells were 11,900 ± 312.05 and 165.67 ± 24.74, respectively, with statistical significance (*P* < 0.05). This result suggested that overexpression of ABCB1 in BJAB/ADR cells could decrease intracellular chemo-drug accumulation by increasing its efflux function.

Verapamil is a known reversal agent against drug resistance that can reverse MDR by blocking the efflux function of ABCB1 without changing its expression level (4, 11). After confirming the efflux function of ABCB1, we further treated BJAB/ADR cells with verapamil to observe the cells' sensitivity to ADR. When BJAB/ADR cells were pretreated with verapamil, the peak fluorescence intensity significantly shifted to the right, and the mean values of the fluorescence intensity increased to 4,890 ± 43.52 (Figure 4Ac). As shown in Figure 4B, verapamil could sensitize the chemoresistance of BJAB/ADR cells to ADR and make BJAB/ADR cells more sensitive to ADR. These results suggested that the resistance mechanism of BJAB/ADR cells might be due to the increased efflux function of ABCB1, and verapamil could mitigate the efflux activity of ABCB1.

### Anti-CD19(Fab)-LDM Had Similar Antitumor Activity in Both Resistant and Parental Cells

**Figure 5:**
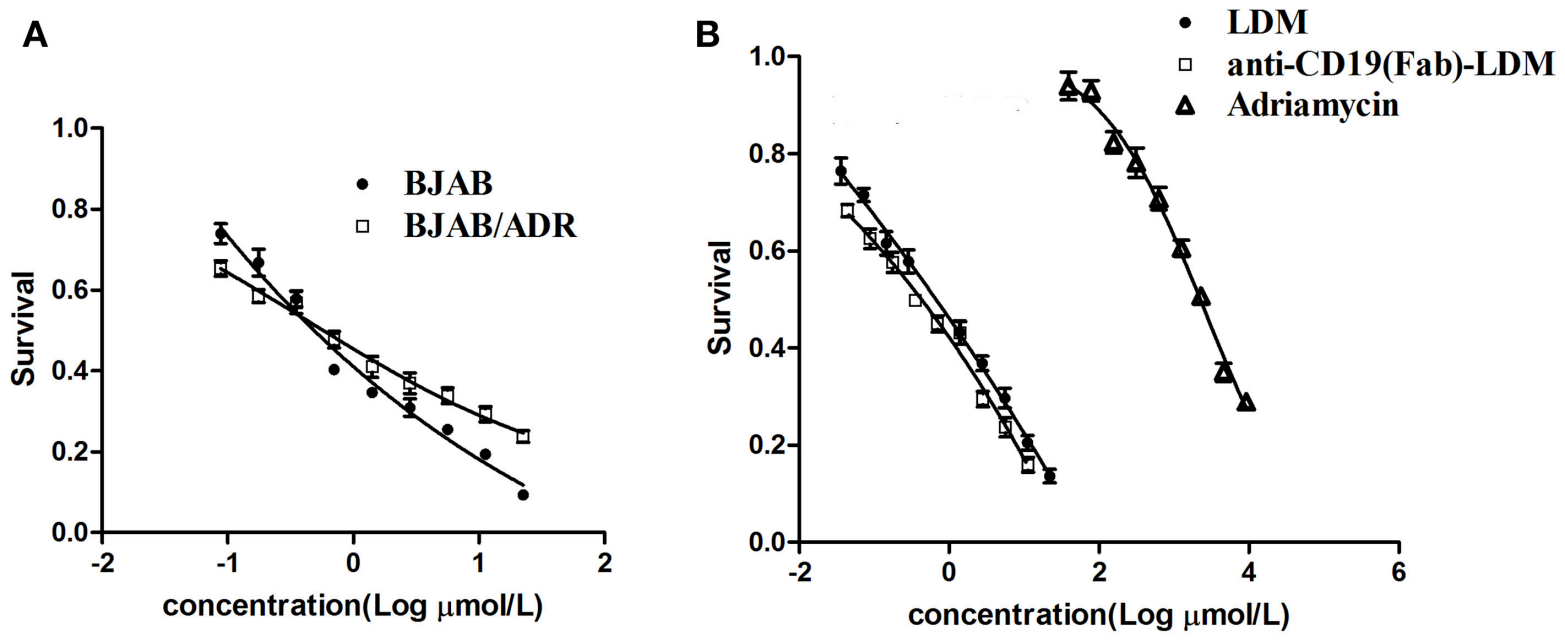
Antitumor activity of anti-CD19(Fab)-LDM on ADR resistant cells. Antitumor activity of anti-CD19(Fab)-LDM on BJAB and BJAB/ADR cells assessed by MTT assay. **(A)** The anti-CD19(Fab)-LDM has a similar cytotoxic effect on resistant cells and their corresponding parental cells. **(B)** The anti-CD19(Fab)-LDM had a stronger inhibitory effect on BJAB/ADR cells than either adriamycin or LDM alone (*P* < 0.05).

Previous experiments in our laboratory showed that the engineered fusion protein anti-CD19(Fab)-LDM exerted significant cytotoxic effects on BJAB cells (23). We performed the MTT assay to ascertain the cytotoxic effect of anti-CD19(Fab)-LDM toward BJAB/ADR cells. As shown in Figure 5A, the growth inhibition curves showed that two types of cells had similar drug sensitivity to anti-CD19(Fab)-LDM (*P* > 0.05). Additionally, the engineered fusion protein anti-CD19(Fab)-LDM showed a much stronger inhibitory effect than ADR in ADR-resistant cells (*P* < 0.01). More importantly, the fusion protein had a stronger cytotoxic effect than LDM alone (Figure 5B). These results suggested that anti-CD19(Fab)-LDM exerted cytotoxic effects on BJAB and BJAB/ADR cells and had a much stronger inhibitory function than either ADR or LDM alone in BJAB/ADR cells.

### Anti-CD19(Fab)-LDM Inhibited Tumor Growth in Both BJAB and BJAB/ADR Xenograft Tumors in BALB/c Nude Mice

We previously demonstrated that anti-CD19(Fab)-LDM suppresses tumor growth in a human B-cell lymphoma xenograft model (23). To assess whether the observed anti-CD19(Fab)-LDM-mediated inhibition of cell growth of MDR cells *in vitro* would extend to animal models, we established BJAB and BJAB/ADR xenograft tumor mouse models to investigate the MDR phenomenon *in vivo* to investigate the therapeutic effect of anti-CD19(Fab)-LDM on the BJAB/ADR xenograft model.

**Figure 6:**
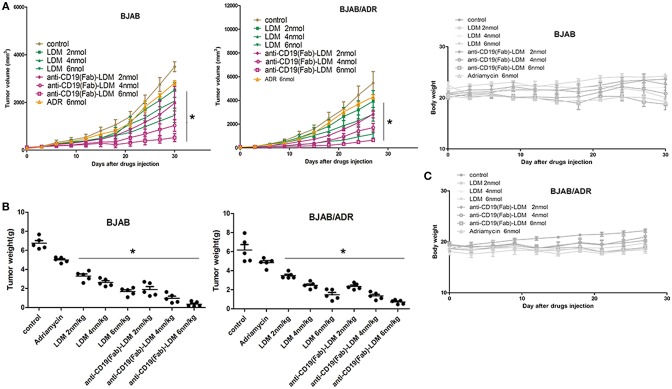
Inhibitory effect of anti-CD19(Fab)-LDM on the growth of xenograft tumors. **(A)** Changes in tumor volume over time in BJAB and BJAB/ADR xenograft models (*n* = 5). Tumor volume was measured once every 3 days. Mice in the anti-CD19(Fab)-LDM groups showed significant inhibition of tumor growth compared with the adriamycin-treated mice in the BJAB and BJAB/ADR xenograft models (^*^P < 0.05 compared to the control). **(B)** Tumor weights of excised BJAB and BJAB/ADR tumor tissues from different mice measured on the 28th day after implantation. The anti-CD19(Fab)-LDM had a significant antitumor effect on both the BJAB and BJAB/ADR xenograft models compared to the effects of adriamycin treatment alone *in vivo*, especially in the high dose group (^*^P < 0.05 compared to the control). **(C)** Body weight of mice with BJAB or BJAB/ADR cell xenografts after treatment. Weight was measured once every 3 days. There was no significant difference in body weight among the groups (*P* > 0.05 compared with control group).

We induced tumors by subcutaneously injecting BJAB or BJAB/ADR cells into the nude mice. When the tumor volume reached 60–80 mm^3^, we treated the mice with PBS (as a control), ADR (6 nmol/kg), LDM (2, 4, or 6 nmol/kg), or anti-CD19(Fab)-LDM (2, 4, or 6 nmol/kg). Tumor volume was measured every 3 days following inoculation. Compared with the LDM- and ADR-treated mice, mice treated with anti-CD19(Fab)-LDM at doses of 2, 4, and 6 nmol/kg showed a significant inhibition of tumor growth in a dose-dependent manner in both the BJAB and BJAB/ADR xenograft models (*P* < 0.05) as shown in Figure 6A. Specifically, the ratio of tumor volume of the anti-CD19(Fab)-LDM group (6 nmol/kg) compared to the PBS control group was 92.79% on day 30, while the inhibitory effect of ADR was 53.45%.

After treatment for 28 days, the tumor tissues were excised and weighed. In the ADR-resistant xenograft model, the antitumor activity of anti-CD19(Fab)-LDM was stronger than that of LDM or ADR alone in a concentration-dependent manner (*P* < 0.05) (Figure 6B). More importantly, anti-CD19(Fab)-LDM was well-tolerated in both the BJAB and BJAB/ADR xenograft models, as indicated by the absence of significant differences in body weight compared with that in the vehicle-treated animals (*P* > 0.05) (Figure 6C). These results suggested that anti-CD19(Fab)-LDM was able to inhibit the growth of ADR-resistant BJAB cells and was well-tolerated. Therefore, anti-CD19(Fab)-LDM could be exploited as a potential drug used in the treatment of multidrug-resistant tumors.

## Discussion

B cell lymphoma is a hematopoietic malignant tumor, and its poor prognosis and short survival are mainly associated with multidrug resistance (MDR). Overcoming MDR and enhance the therapeutic effect of regimens for the treatment of B cell lymphoma is a major concern in clinical oncology (28–30). Thus, there is an immediate need to identify novel targets for the treatment of B cell leukemias and lymphomas. It was known that the poor response of lymphoma to chemotherapeutic drugs is mainly due to acquired MDR rather than innate resistance (31–33). Therefore, an appropriate experimental model is urgently needed for the study of MDR in B cell lymphoma. Since Bielder and Riehm first reported the MDR phenomenon of tumor cells in 1970, a series of multidrug-resistant cell lines have been constructed. However, there is few report on the stable MDR cell line of B cell lymphoma (34). In this article, our laboratory successfully established a B lymphoma MDR cell line, named BJAB/ADR, with the first-line chemotherapeutic drug adriamycin (ADR). The resistance factor (RF) between the parental and resistant cell lines was 43-fold. In fact, the resistance fold is highly variable between cell lines. For example, Wattanawongdon established two gemcitabine-resistant human cholangiocarcinoma cell lines with resistance indices of approximately 25- and 62-fold, respectively (35). In contrast, Iwasaki developed a cisplatin-resistant human neuroblastoma cell line with a resistance variant of approximately 1.1 (36). Generally, medium resistance is the most common type encountered in clinical practice. It is worth mentioning that BJAB/ADR cells could stably grow in drug-free medium for several weeks, and the morphological characteristics are consistent with those of parental cells, indicating a resistance-mediated improvement in survival. However, BJAB/ADR cells prefer to grow in clusters and have a slower growth rate than its parental cells (Figures 1, 2). More importantly, these cells exhibited cross-resistance to a variety of structurally and functionally unrelated antineoplastic agents, such as etoposide, daunorubicin, homoharringtonine and mitoxantrone (Table 2). This result provides important information for further clinical evaluation.

We firstly examined the cell cycle of BJAB and BJAB/ADR. The results showed that ADR could induce G0/G1 phase arrest in BJAB/ADR cells compared with that in BJAB cells (Table 1). Combined the results of growth rate in Figure 2, we postulated that ADR could induce longer proliferation time and poorer proliferation ability. However, the difference of phase distribution between BJAB and BJAB/ADR was not obvious. Hence, it is needed more further studies to figure out the exact resistance mechanism of ADR on BJAB/ADR cell line. Also, we hypothesized that ADR resistance is probably associated with the overexpression of ABC transporters. This hypothesis is supported by the cross-resistance to other structurally unrelated chemotherapeutic drugs, most of which are substrates of ABC transporters, in resistant cells. ABCB1 (P-gp), ABCG2 (BCRP), and ABCC1 (MRP1) are three ABC transporters that are broadly expressed in multidrug-resistant cell lines (37). Thus, we examined the expression of these three ABC transporters and found that the *ABCB1* gene and P-gp protein expression was significantly upregulated in the BJAB/ADR cells. In contrast, the *ABCC1* and *ABCG2* mRNA and protein levels were only slightly increased compared to ABCB1 (Figure 3). This result is consistent with the previous reports that upregulated *ABCB1* gene is the main response for MDR in B-cell lymphoma (38, 39). Moreover, the Rhodamine 123 (Rho 123) exclusion assay verified that overexpression of ABCB1 participated in MDR, and verapamil, a known ABCB1 inhibitor, could reverse this drug resistance, thus increasing the sensitivity of BJAB/ADR cells to ADR (Figure 4). According to the present results, we could conclude that ABCB1-overexpressing is responsible for chemoresistance in BJAB/ADR cell line and poor efficacy of chemotherapeutic agents.

About 80–90% of cases of non-Hodgkin lymphoma (NHL) are of B-cell origin (40). The current therapeutic approach for B cell lymphoma involves chemotherapy, radiotherapy and the incorporation of the anti-CD20 monoclonal antibody rituximab (41, 42). Chemotherapy is the most common treatment strategy, but the outcomes of patients are often very poor, because of the development of resistance to conventional chemotherapeutic strategies. To overcome this issue, chemo-immunotherapies using rituximab in combination with CHOP (refers to cyclophosphamide, doxorubicin, vincristine, and prednisone) (R-CHOP) have markedly improved the outcome of patients with B cell lymphoma in recent decades. Currently, there are some novel treatment regimes, such as bendamustine or valproate, in combination with R-CHOP for patients with different phases of lymphoma (43–45). Unfortunately, about 10–15% of patients fail to respond to R-CHOP treatment, and 20–25% of patients develop relapse (46, 47). Therefore, novel strategies are needed to improve patients' response rate. Lidamycin is a novel antibiotic with antitumor activity emerged in recent years. Its mechanism of antineoplastic action is to inhibit DNA synthesis and break down cellular DNA in carcinoma cells (16). Due to its unique structure, lidamycin is often reconstituted with antibodies to establish engineered fusion proteins to maintain both the target property of antibodies and the cytotoxic effect. This type of biopharmaceutical drug is called antibody-drug conjugate (ADC) (48). Specifically, anti-C19(Fab)-LDM is an engineered fusion protein previously established in our laboratory and has been reported to have high antineoplastic activity toward B cell lymphoma (23). The fusion protein anti-CD19(Fab)-LDM was developed as a targeted therapy for lymphoma and induces significant tumor-specific cytotoxicity. Thus, anti-CD19(Fab)-LDM can overcome the deficiencies of traditional chemotherapy agents and significantly decrease adverse effects in patients. Additionally, this study shed light on the solution of drug resistance in tumor treatment. With all of these advantages, the use of engineered fusion proteins can circumvent the clinical issue of chemotherapy in the treatment of lymphoma. More importantly, due to the strong cytotoxic effects of LDM, the antibody-drug conjugate anti-CD19(Fab)-LDM can be administered at a lower dose to achieve therapeutic effects. Thus, it is a novel strategy worth exploring to find out its promising potential in preclinical and clinical trials.

Considering the strong antitumor activity of LDM and the B cell-targeted property of the anti-C19(Fab) antibody, we postulated that the anti-C19(Fab)-LDM could exert cytotoxic effect on the resistant cells of B cell lymphoma. As expected, anti-C19(Fab)-LDM showed similar cytotoxic effects toward BJAB/ADR and BJAB cells and showed a greater effect than either LDM or ADR alone (Figure 5). From the *in vivo* results, anti-C19(Fab)-LDM exhibited more potent antitumor activities than LDM and ADR in the BJAB/ADR xenograft mouse model (Figure 6). The *in vivo* results were in consistent with the *in vitro* results. Importantly, the therapeutic effect of anti-C19(Fab)-LDM was better than that of LDM both *in vitro* and *in vivo*. Therefore, our current results indicated that anti-C19(Fab)-LDM could be a promising targeted therapy for patients with ADR-resistant B cell lymphoma. Considering our *in vitro* results above, it is reasonable to postulate that anti-C19(Fab)-LDM may have inhibitory effect to pumped function of ABCB1, in turn probably increase the intracellular concentration of antineoplastic drugs. However, the exactly underlying re-sensitive mechanism of anti-C19(Fab)-LDM is needed to be addressed in the future.

In summary, we established an MDR B cell lymphoma cell line named BJAB/ADR, which could represent a drug-resistant cell model for lymphoma research. Additionally, our previously developed engineered fusion protein anti-C19(Fab)-LDM can be used to overcome MDR for the treatment of B cell lymphoma, especially in patients with acquired ADR resistance.

## Data Availability

The raw data supporting the conclusions of this manuscript will be made available by the authors, without undue reservation, to any qualified researcher.

## Author Contributions

DF, LJ, MY, and DX designed the experiments. DF, LJ, YS, and SB performed experiments. YY, XY, and YZ provided technical and material support. DF and LJ wrote the first draft. DF, LJ, and MY revised the manuscript. All authors discussed the results and implications and developed the manuscript at all stages.

### Conflict of Interest Statement

The authors declare that the research was conducted in the absence of any commercial or financial relationships that could be construed as a potential conflict of interest.
